# Comparison of functional properties of unripe papaya fruits of different sexes

**DOI:** 10.5511/plantbiotechnology.24.0421a

**Published:** 2024-06-25

**Authors:** Kota Kera, Haruka Asada, Shunsuke Kikuchi, Shoma Saito, Masumi Iijima, Tsutomu Nakayama, Hideyuki Suzuki

**Affiliations:** 1 Department of Nutritional Science and Food Safety, Faculty of Applied Bioscience, Tokyo University of Agriculture; 2 Department of Research and Development, Kazusa DNA Research Institute

**Keywords:** *Carica papaya* L., carpaine, dehydrocarpaine, metabolome analysis, sex

## Abstract

Papaya (*Carica papaya* L.) is a herbaceous plant belonging to the family Caricaceae in the order Brassicales. The shape of papaya fruit was linked to sex, and the fruit of female plants is round, whereas that of hermaphrodites is pyriform. Although fruit shape preferences vary by region, differences in their functionalities have not been investigated. Since unripe fruit, also called green papaya, is known for its nutritional and therapeutic benefits, we performed a metabolome analysis of unripe papaya using liquid chromatography coupled with quadrupole/time of flight mass spectrometry. We first focused on capraine derivatives, major piperidine alkaloids, and bioactive compounds with significant antiplasmodial activity. Interestingly, carpaine derivatives tended to be altered in the peel and pulp but not in the seed. Multivariate analyses indicated little difference or minor differences to the extent that they can be caused by individual differences in metabolite profiling between the two sexes. Conversely, total polyphenol content and proteolytic activity were also investigated, but there were no differences between females and hermaphrodites for total polyphenol content and proteolytic activity. In conclusion, the metabolome and major functionalities were similar between hermaphrodites and female unripe fruit. However, it would be worth considering the sex of the material fruit, especially when focusing on the functional properties of carpaine derivatives.

Papaya (*Carica papaya* L.) is a herbaceous plant belonging to the family Caricaceae in the order Brassicales and is widely cultivated in tropical and subtropical countries. Papaya cultivars are trioecious with three sexes: male, female, and hermaphrodite, which are determined by monogenic inheritance involving three alleles (Aryal and Ming 2014; Avila-Hernandez et al. 2023; Chan and Sim 2019). The dominant alleles are *M* for males and *M^h^* for hermaphrodites, and the recessive allele is *m* for females. Since all homozygous dominants (*MM*, *MM^h^*, and *M^h^M^h^*) are embryonic lethal, male (*Mm*) and hermaphrodite (*M^h^m*) are heterozygotes while female (*mm*) is a homozygous recessive, resulting in a 2 : 1 segregation of hermaphrodite to female from self-pollinated hermaphrosite seeds and a 1 : 1 segregation of hermaphrodite to female from cross-pollinated female seeds (Ming et al. 2007). Interestingly, the papaya fruit is sex-linked, and the fruit of female plants is round, whereas that of hermaphrodites is pyriform in shape (Figure 1). Although fruit shape preferences vary by region, the reason seems to be impression and processing convenience, not eating quality or total soluble solid content (Chan and Sim 2019). The unripe fruit, also called green papaya, is known for its nutritional and therapeutic benefits (Ikram et al. 2015) and proteolytic activity (Esti et al. 2013). Moreover, we proposed the worth of the underutilized peel of unripe papaya as a source of functional materials in the food and pharmaceutical industries (Hiraga et al. 2021). Here, we performed metabolome analysis using liquid chromatography coupled with quadrupole/time of flight mass spectrometry (LC/Q-TOF) and functionalities in total polyphenol content and proteolytic activity of hermaphrodite (*M^h^m*) and female (*mm*) fruit to investigate the difference in value as a functional food.

**Figure 1:**
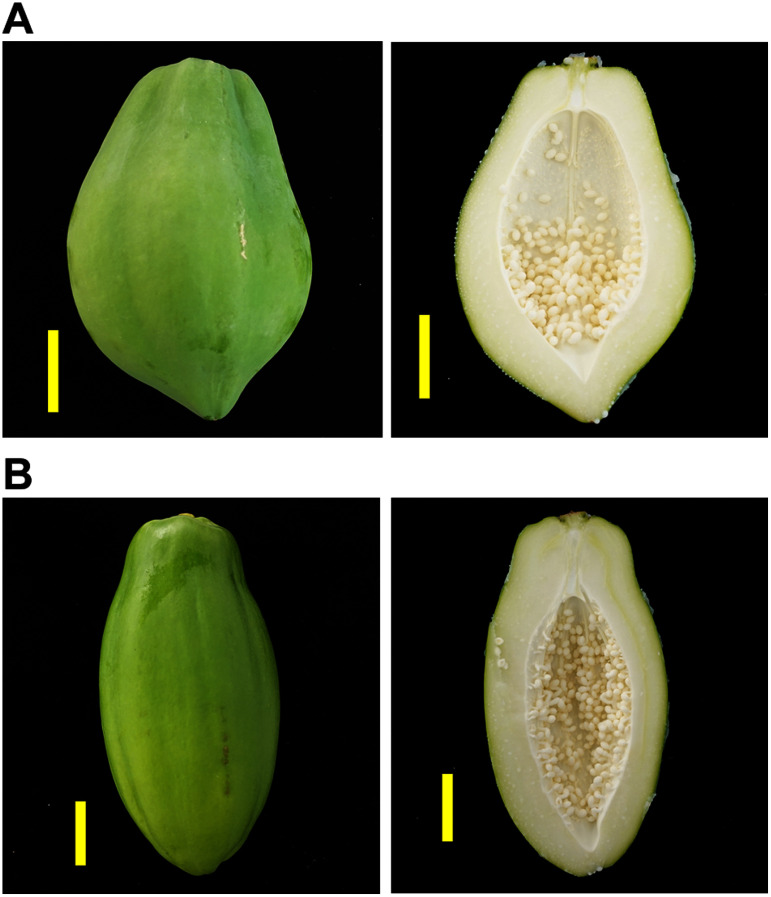
Figure 1. Photographs of papaya fruits. A. Whole (left panel) and sliced (right panel) female unripe papaya were used for analysis. B. Whole (left panel) and sliced (right panel) hermaphrodite unripe papaya were used for analysis. Scale bars: 5 cm.

Four hermaphrodites and female unripe papaya fruits were obtained from Yaginuma Farm Co., Ltd. (Ibaraki, Japan) in mid-October 2021. They were separated into peel, pulp, and seed and stored at −80°C until use. The stored samples were lyophilized and powdered using a milser (IFM-800DGM; Iwatani Corporation, Osaka, Japan). Subsequently, the powder (10 mg) was mixed with 500 µl of 80% methanol, centrifuged at 15,000 min^−1^ for 10 min, and filtered using a Mono-Spin C18 column (GL Science Inc., Tokyo, Japan). The eluted solutions were filtered through a 0.2 µm polytetrafluoroethylene (PTFE) membrane (Merck Millipore, MA, USA) and analyzed by LC/Q-TOF (Agilent 6530 Accurate Mass Q-TOF, Agilent Technologies, Inc., CA, USA). The concentration of samples were adjusted according to the measured dry-wight of powder. Following dilution (peel, ×0.05; pulp, not diluted; seed, ×0.5), the sample solution (1 µl) was injected into an InertSustain AQ-C18 (column size: 2.1×150 mm; particle size: 3.0 µm; GL Science Inc.). Mobile phases A (0.1% formic acid in water) and B (0.1% formic acid in acetonitrile) were used with a gradient of 2% B for 0–3 min, 2–98% B for 3–30 min, 98% B for 30–35 min, and 2% B for 35–40 min. Column temperature and flow rate were maintained at 40°C and 200 µl min^−1^, respectively. Mass spectrometry analysis was performed in electrospray ionization positive mode, covering a mass range from *m*/*z* 50 to 1,500 for full mass scans and *m*/*z* 25 to 1,500 for the targeted MS/MS scans. An automated processing program based on ProteoWizard (Chambers et al. 2012) and PowerGet (Sakurai et al. 2014) was used for peak detection, characterization, and alignment. Metabolite annotation was performed using MFSearcher (Sakurai et al. 2013) with a mass accuracy of 5 ppm. Thus, peak lists for peel, pulp, and seed consisting of 218, 462, and 478 peaks, respectively, were generated (Supplementary Data S1). To investigate the difference, we first focused on capraine derivatives (Figure 2A), which are major piperidine alkaloids (Julianti et al. 2014b) that are specifically detected in the Caricaceae family according to KNApSAcK database (Afendi et al. 2012). Carpaine is a primary bioactive compound with significant antiplasmodial activity, whereas its putative composition unit, carpamic acid, did not show any activity (Julianti et al. 2014a). Each putative ion peak was annotated by MS/MS spectral data (Supplementary Data S2) as described previously (Hiraga et al. 2021). Their peak area (carpaine: *m*/*z* 240.1958±100 ppm [M+2H]^2+^; dehydrocarpaine I: *m*/*z* 477.3687±10 ppm [M+H]^+^; dehydrocarpaine II: *m*/*z* 475.3530±10 ppm [M+H]^+^; carpamic acid: *m*/*z* 258.2064±20 ppm [M+H]^+^; dehydrocarpamic acid: *m*/*z* 256.1907±20 ppm [M+H]^+^) was acquired using MassHunter software version B.05.01 (Agilent Technologies, Inc.) (Figure 2B, C, D). Although there were no significant differences, carpaines in females tended to be higher than those in hermaphrodites for peel and pulp. Dehydrocarpaine I in females was higher than in hermaphrodites for peel and pulp but not for seed. Dehydrocarpaine II in females was trended to be higher for pulp than in hermaphrodites. Dehydrocarpamic acid in the peel of females was lower than that in hermaphrodites. When taken together, some carpaine derivatives were altered in the peel and pulp but not in the seed.

**Figure 2:**
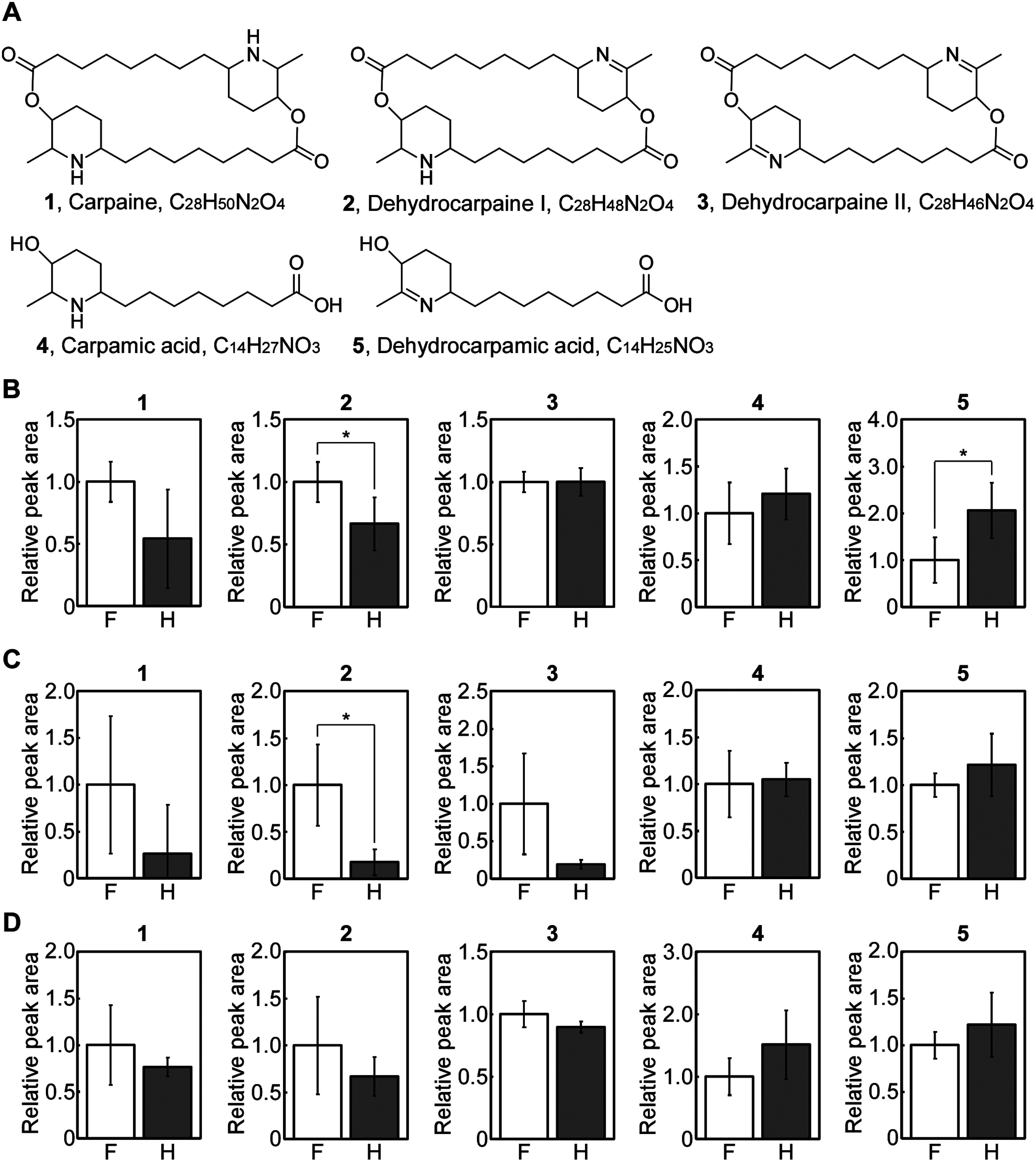
Figure 2. Putative carpaine derivatives content in unripe papaya extracts. A. Chemical structures of carpaine derivatives. B–D. The relative peak area of each compound in peel (B), pulp (C), and seed (D) from female (F) and Hermaphrodite (H) trees. Values represent the mean±S.D. of four biological replicates. The asterisk indicates a significant difference according to the Welch *t*-test (*p*<0.05). The numbers above the graph correspond to the carpaine derivatives.

Multivariate analyses were performed with Pareto scaling using MetaboAnalyst 5.0 (Pang et al. 2021) to provide an overview of the metabolite profiling of hermaphrodites and female unripe papaya fruits. The principal component analysis (PCA) score plot showed that the 95% confidence region of the hermaphrodites partially overlapped with that of the females in all parts (Figure 3A). Moreover, hierarchical clustering analysis (HCA) suggested that hermaphrodites and females were partially distinguishable, but some individuals of each sex belonged to the same cluster (Figure 3B). These results indicated that there were little differences or minor differences to the extent that they can be caused by individual differences in metabolite profiling between the two sexes.

**Figure 3:**
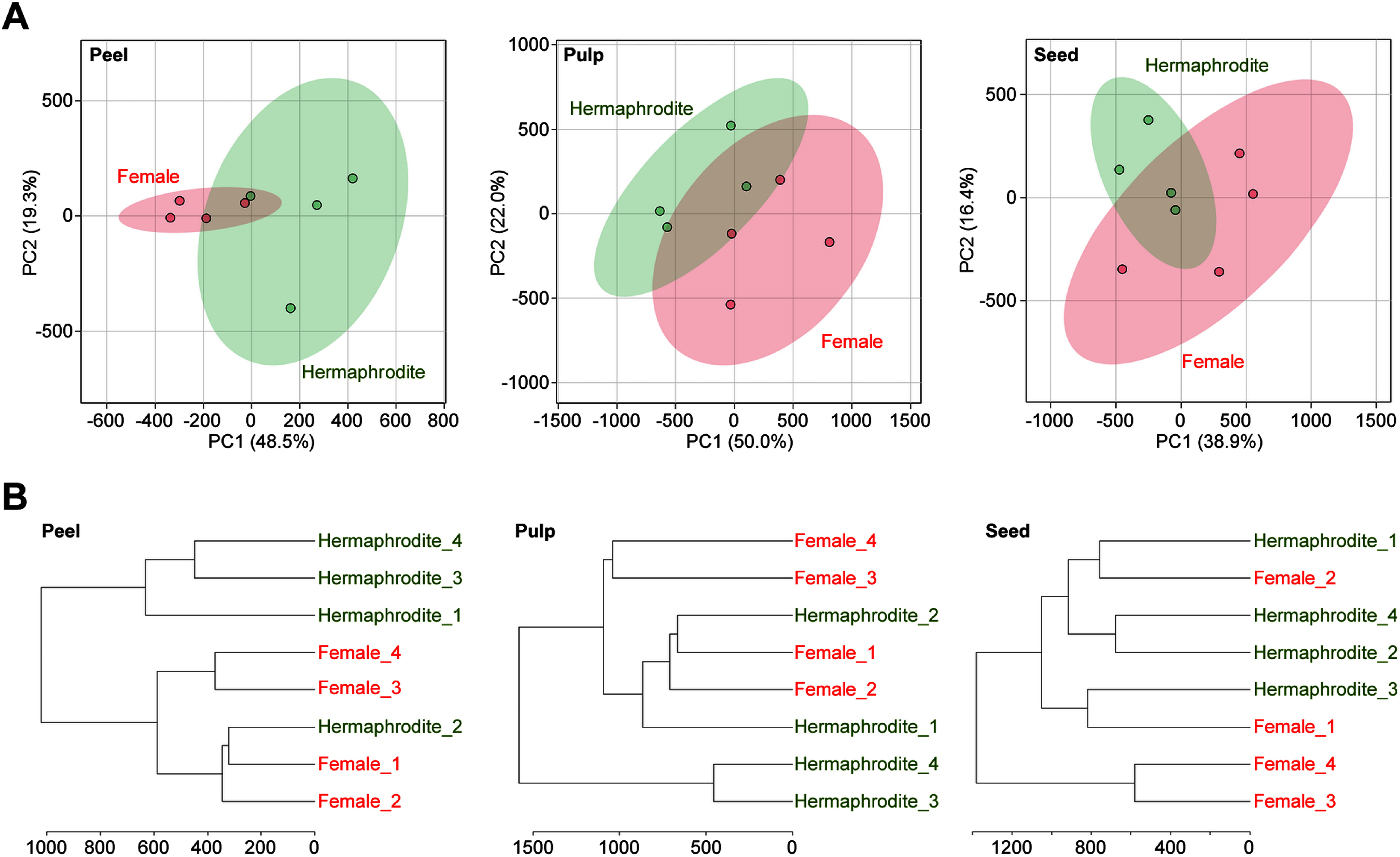
Figure 3. Multivariate analysis of metabolite profiles derived from female and hermaphrodite unripe papaya fruit. A. The principal component analysis (PCA) score plots. Each colored ellipse represents the 95% confidence region. B. The hierarchical clustering analysis (HCA) dendrogram. The conditions were as follows: distance, Euclidean; clustering algorithm, Ward.

Finally, we investigated the other functionalities of total polyphenol content and proteolytic activity. Total polyphenol content was measured using the Folin–Ciocalteu method as described previously, with some modifications (Hiraga et al. 2021). Details are provided in Supplementary Data S3. There were no differences between females and hermaphrodites in any part (Supplementary Data S3). Proteolytic activity was measured using casein as a substrate, as described previously, with some modifications (Hiraga et al. 2021). Details are provided in Supplementary Data S4. The specific activity of the peel was much higher than that of the pulp and seed. However, there were no differences between females and hermaphrodites in any part (Supplementary Data S4).

In conclusion, the metabolome and major functionalities were similar between hermaphrodites and female unripe fruit. However, it would be worth considering the sex of the material fruit, especially when focusing on the functional properties of carpaine derivatives.

## Abbreviations

HCA: hierarchical clustering analysis
LC/Q-TOF: liquid chromatography coupled with quadrupole/time of flight mass spectrometry
PCA: principal component analysis

## Data deposition

The raw datasets were deposited into the MetaboBank metabolome database under the accession numbers MTBKS235 and are available for free download.

## Description of Supplementary Files

Supplementary Data S1: Peak lists for peel, pulp, and seed.

Supplementary Data S2: MS/MS spectra.

Supplementary Data S3: Metarials and methods and result for measurment of total polyphenol content.

Supplementary Data S4: Metarials and methods and result for measurment of proteolytic activity.
