# Supplementary material for: Comparison of functional properties of unripe papaya fruits of different sexes
Source: Plant Biotechnol (Tokyo). 2024 Jun 25;41(2):165–8. doi: 10.5511/plantbiotechnology.24.0421a (PMC11500600; doi:10.5511/plantbiotechnology.24.0421a)
Supplement: Supplementary Data [file plantbiotechnology-41-2-24.0421a-s001.pdf]

Supplementary Data S2

Carpaine, putative ($C_{28}H_{50}N_2O_4$)

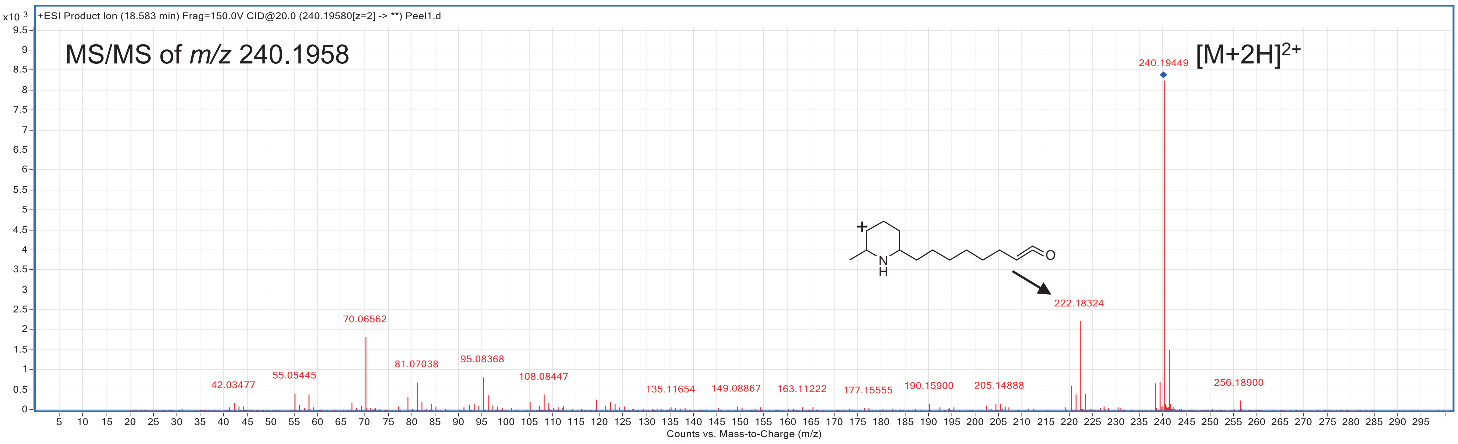

Dehydrocarpaine I, putative ($C_{28}H_{48}N_2O_4$)

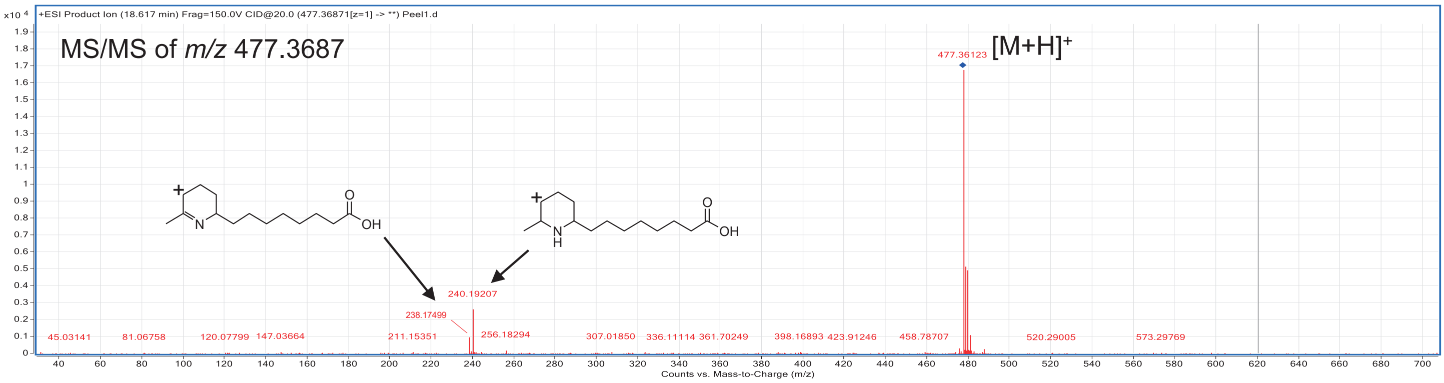

Dehydrocarpaine II, putative ($C_{28}H_{46}N_2O_4$)

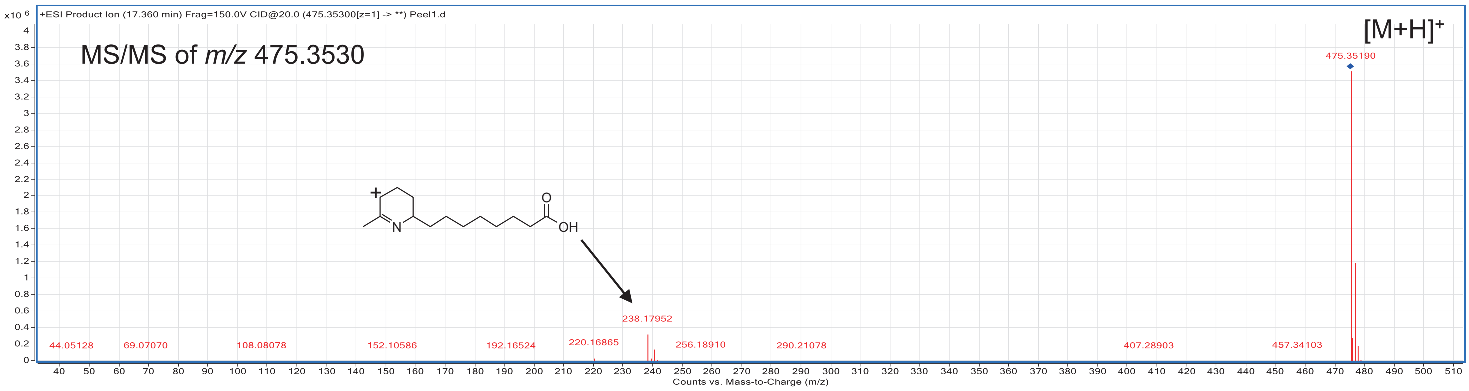

Carpaimic acid, putative ($C_{14}H_{27}NO_3$)

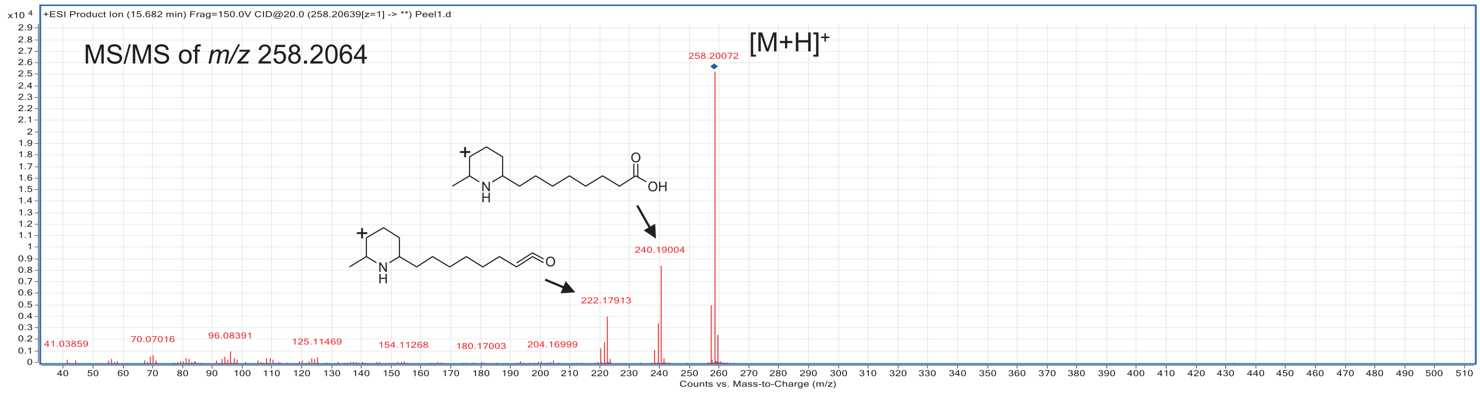

Dehydrocarpamic acid, putative ($C_{14}H_{25}NO_3$)

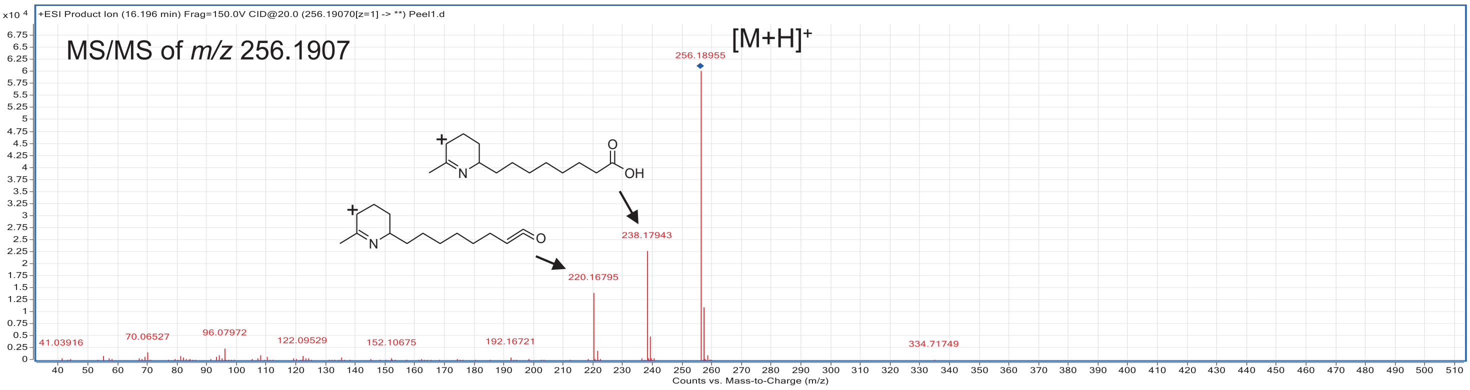

Supplementary Data 3

Materials and methods and result for measurement of total polyphenol content

Comparison of functional properties of unripe papaya fruits of different sexes

Kota Kera[1 ¶], Haruka Asada[1], Shunsuke Kikuchi[1], Shoma Saito[1], Masumi Iijima[1], Tsutomu Nakayama[1], Hideyuki Suzuki[2]

[1] Department of Nutritional Science and Food Safety, Faculty of Applied Bioscience, Tokyo University of Agriculture, 1-1-1 Sakuragaoka, Setagaya-ku, Tokyo 156-8502, Japan
[2] Department of Department of Applied Genomics, Kazusa DNA Research Institute, 2-6-7 Kazusa-Kamatari, Kisarazu, Chiba 292-0818, Japan

Materials and methods

The dried fruit powder of unripe papaya was resuspended in 500 mM phosphate buffer and incubated for 30 minutes at 4 °C. The supernatant was collected as the sample solution after centrifugation at 18,000 × g for 5 min at 4 °C. The sample solution (100 µl) was mixed with 500 µl of 10 % Folin-Ciocalteu reagent (FUJIFILM Wako Pure Chemical Corporation, Tokyo, Japan) and 400 µl of 7.5 % Na_2CO_3 (Kumazawa *et al.* 2002). After vortexing and incubation for 1 hour at 25 °C, the absorbance was measured at 765 nm using a spectrophotometer (U-2900, Hitachi High-Tech Science Corporation, Tokyo, Japan).

References

Kumazawa S, Taniguchi M, Suzuki Y, Shimura M, Kwon MS, Nakayama T (2002) Antioxidant activity of polyphenols in carob pods. *J Agric Food Chem* 50: 373–377

Data

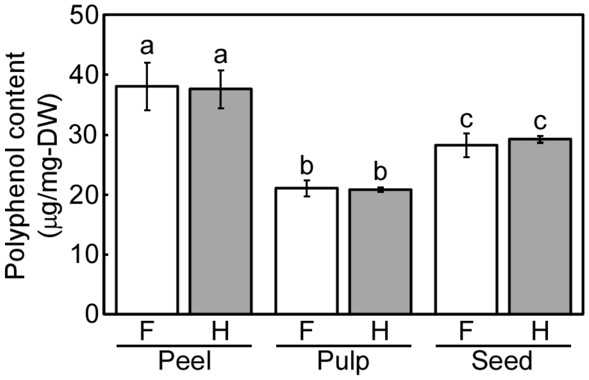

Supplementary Figure S3. Polyphenol content in unripe papaya extracts.

Each part of the fruit is from the female (F) and Hermaphrodite (H) tree. Values represent mean ± S.D. of four biological replicates from three technical replicates. According to Tukey's test, clusters with identical letter codes did not exhibit significant differences ($p < 0.05$).

Supplementary Data 4

Materials and methods and results for measurement of proteolytic activity

Comparison of functional properties of unripe papaya fruits of different sexes

Kota Kera[1 ¶], Haruka Asada[1], Shunsuke Kikuchi[1], Shoma Saito[1], Masumi Iijima[1], Tsutomu Nakayama[1], Hideyuki Suzuki[2]

[1] Department of Nutritional Science and Food Safety, Faculty of Applied Bioscience, Tokyo University of Agriculture, 1-1-1 Sakuragaoka, Setagaya-ku, Tokyo 156-8502, Japan

[2] Department of Department of Applied Genomics, Kazusa DNA Research Institute, 2-6-7 Kazusa-Kamatari, Kisarazu, Chiba 292-0818, Japan

Materials and methods

The dried fruit powder of unripe papaya was resuspended in 500 mM phosphate buffer and incubated for 30 minutes at 4 °C. Following centrifugation at 18,000 × *g* for 5 min at 4 °C, the supernatant was collected and filtered with 0.45 µm polyvinylidene difluoride membrane (Merck Millipore, MA, USA). Proteolytic activity was measured using casein as a substrate, as described previously, with some modifications (Hiraga *et al.* 2021). The sample solution (20 µl) was mixed with 2.5 µl of 80 mM cysteine solution and 2.5 µl of 40 mM ethylenediaminetetraacetic acid disodium salt solution. After vortexing and incubation for 5 min at 38 °C, the reaction was initiated by adding 25 µl of 1 % (w/v) casein solution. The reaction was terminated by adding 75 µl of 5 % (w/v) cold trichloroacetic acid solution. The supernatant was centrifuged at 9,000 × *g* for 10 min at 4 °C, and absorbance was measured at 275 nm using a spectrophotometer (U-2900, Hitachi High-Tech Science Corporation, Tokyo, Japan). One unit of proteolytic activity was defined as the enzyme required to increase the absorbance at 275 nm by 0.01 per minute at pH 7.0 and 38 °C. The protein concentration of the sample solution was measured using TaKaRa Bradford Protein Assay Kit (Takara Bio Inc., Shiga, Japan) and a portable photo absorbance meter (PiCOEXPLORER, Yamato Scientific Co., Ltd., Tokyo, Japan).

References

Hiraga Y, Ara T, Sato N, Akimoto N, Sugiyama K, Suzuki H, Kera K (2021) Metabolic analysis of unripe papaya (Carica papaya L.) to promote its utilization as a functional food. *Bioscience, Biotechnology, and Biochemistry*:

Data

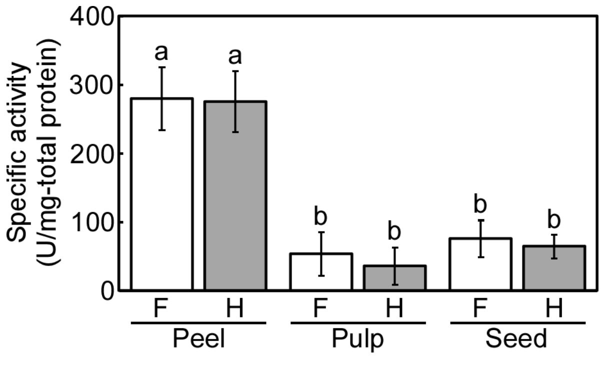

Supplementary Figure S4. Proteolytic activity in unripe papaya extracts.

Each part of the fruit is from a female (F) and Hermaphrodite (H) tree. Values represent mean ± S.D. of four biological replicates from three technical replicates. According to Tukey's test, clusters with identical letter codes did not exhibit significant differences ($p < 0.05$).
